# Supplementary material for: Causal relationship between COVID-19 and chronic pain: A mendelian randomization study
Source: PLoS One. 2024 Jan 19;19(1):e0295982. doi: 10.1371/journal.pone.0295982 (PMC10798446; doi:10.1371/journal.pone.0295982)
Supplement: S1 Table — (DOCX) [file pone.0295982.s001.docx]

**Supplemental Table 1. Detailed information pertaining to SNPs in the two-sample MR analysis.**

| Exposure | Outcome | Number of SNPs | | | Removed due to harmonization | Filtering for snp significantly associated with exposure in outcome data |
| --- | --- | --- | --- | --- | --- | --- |
|  |  | Extracted from exposure | Extracted from outcomes | After harmonising with outcome |  |  |
| COVID-19 (infection vs. normal population) | Pain in joint | 45 | 14 | 11 | rs2126342,rs6583441,rs6911758 | NA |
|  | Pain in joint (Lower leg) | 45 | 15 | 12 | rs2126342,rs6583441,rs6911758, | NA |
|  | Low back pain | 45 | 20 | 17 | rs2126342,rs6583441,rs6911758, | NA |
|  | Low back pain (Lumbar region) | 45 | 14 | 11 | rs2126342,rs6583441,rs6911758, | NA |
|  | Pain in limb (Lower leg) | 45 | 15 | 12 | rs2126342,rs6583441,rs6911758, | NA |
|  | Back pain | 45 | 43 | 40 | rs2126342,rs6583441,rs6911758, | NA |
|  | Facial pain | 45 | 30 | 27 | rs2126342,rs6583441,rs6911758, | NA |
|  | Headache | 45 | 43 | 40 | rs2126342,rs6583441,rs6911758, | NA |
|  | Hip pain | 45 | 43 | 40 | rs2126342,rs6583441,rs6911758, | NA |
|  | Knee pain | 45 | 43 | 40 | rs2126342,rs6583441,rs6911758, | NA |
|  | Neck or shoulder pain | 45 | 43 | 40 | rs2126342,rs6583441,rs6911758, | NA |
|  | Stomach or abdominal pain | 45 | 43 | 40 | rs2126342,rs6583441,rs6911758, | NA |
|  | Pain all over the body | 45 | 42 | 39 | rs2126342,rs6583441,rs6911758, | NA |
| COVID-19 (hospitalized vs. normal population) | Pain in joint | 41 | 13 | 12 | rs2282578, | NA |
|  | Pain in joint (Lower leg) | 41 | 15 | 14 | rs2282578, | NA |
|  | Low back pain | 41 | 17 | 16 | rs2282578, | NA |
|  | Low back pain (Lumbar region) | 41 | 13 | 12 | rs2282578, | NA |
|  | Pain in limb (Lower leg) | 41 | 15 | 14 | rs2282578, | NA |
|  | Back pain | 41 | 39 | 38 | rs2282578, | NA |
|  | Facial pain | 41 | 27 | 26 | rs2282578, | NA |
|  | Headache | 41 | 38 | 37 | rs2282578, | NA |
|  | Hip pain | 41 | 39 | 38 | rs2282578, | NA |
|  | Knee pain | 41 | 39 | 38 | rs2282578, | NA |
|  | Neck or shoulder pain | 41 | 39 | 38 | rs2282578, | NA |
|  | Stomach or abdominal pain | 41 | 39 | 38 | rs2282578, | NA |
|  | Pain all over the body | 41 | 40 | 39 | rs2282578, | NA |
| COVID-19 (hospitalized vs. not hospitalized) | Pain in joint | 28 | 5 | 5 | NA | NA |
|  | Pain in joint (Lower leg) | 28 | 5 | 5 | NA | NA |
|  | Low back pain | 28 | 8 | 8 | NA | NA |
|  | Low back pain (Lumbar region) | 28 | 5 | 5 | NA | NA |
|  | Pain in limb (Lower leg) | 28 | 6 | 6 | NA | NA |
|  | Back pain | 28 | 26 | 26 | NA | NA |
|  | Facial pain | 28 | 18 | 18 | NA | NA |
|  | Headache | 28 | 26 | 25 | NA | rs7225002, |
|  | Hip pain | 28 | 26 | 26 | NA | NA |
|  | Knee pain | 28 | 26 | 26 | NA | NA |
|  | Neck or shoulder pain | 28 | 26 | 26 | NA | NA |
|  | Stomach or abdominal pain | 28 | 26 | 26 | NA | NA |
|  | Pain all over the body | 28 | 26 | 26 | NA | NA |
| COVID-19 (very severe respiratory confirmed vs. normal population) | Pain in joint | 51 | 16 | 16 | NA | NA |
|  | Pain in joint (Lower leg) | 51 | 19 | 19 | NA | NA |
|  | Low back pain | 51 | 23 | 23 | NA | NA |
|  | Low back pain (Lumbar region) | 51 | 15 | 15 | NA | NA |
|  | Pain in limb (Lower leg) | 51 | 19 | 19 | NA | NA |
|  | Back pain | 51 | 48 | 48 | NA | NA |
|  | Facial pain | 51 | 37 | 37 | NA | NA |
|  | Headache | 51 | 48 | 47 | NA | rs7080472, |
|  | Hip pain | 51 | 48 | 48 | NA | NA |
|  | Knee pain | 51 | 48 | 48 | NA | NA |
|  | Neck or shoulder pain | 51 | 48 | 48 | NA | NA |
|  | Stomach or abdominal pain | 51 | 48 | 48 | NA | NA |
|  | Pain all over the body | 51 | 23 | 23 | NA | NA |

MR, Mendelian randomization; SNP, single-nucleotide polymorphism.
