# Supplementary material for: Causal relationship between COVID-19 and chronic pain: A mendelian randomization study
Source: PLoS One. 2024 Jan 19;19(1):e0295982. doi: 10.1371/journal.pone.0295982 (PMC10798446; doi:10.1371/journal.pone.0295982)
Supplement: S2 Table — (DOCX) [file pone.0295982.s002.docx]

**Supplemental Table 2. MR estimates of associations between COVID-19 (infection vs. normal population) and bodily pain in various regions across different methods**

| Trait | IVW | | | MR Egger | | | Weighted median | | | Heterogeneity test | | | | Pleiotropy test | |
| --- | --- | --- | --- | --- | --- | --- | --- | --- | --- | --- | --- | --- | --- | --- | --- |
|  | **β** | se | p | **β** | se | p | β | se | p | IVW Q | p | MR‐Egger Q | p | MR‐Egger *p* | PRESSO *p* |
| Pain in joint | 1.38E-04 | 6.16E-04 | 8.22E-01 | 9.54E-04 | 2.32E-03 | 6.90E-01 | 2.84E-04 | 8.28E-04 | 7.32E-01 | 9.21E+00 | 5.12E-01 | 9.08E+00 | 4.30E-01 | 7.23E-01 | 3.70E-01 |
| Pain in joint (Lower leg) | 9.10E-04 | 7.05E-04 | 1.96E-01 | 1.12E-03 | 2.86E-03 | 7.03E-01 | 5.67E-04 | 8.85E-04 | 5.21E-01 | 1.38E+01 | 2.47E-01 | 1.37E+01 | 1.85E-01 | 9.40E-01 | 4.48E-01 |
| Low back pain | 1.23E-04 | 6.74E-04 | 8.55E-01 | 8.73E-04 | 2.87E-03 | 7.65E-01 | -3.81E-04 | 9.37E-04 | 6.84E-01 | 9.43E+00 | 8.95E-01 | 9.35E+00 | 8.58E-01 | 7.92E-01 | 6.66E-01 |
| Low back pain (Lumbar region) | -6.99E-04 | 6.10E-04 | 2.52E-01 | 4.42E-04 | 2.29E-03 | 8.51E-01 | -2.48E-04 | 8.28E-04 | 7.64E-01 | 7.50E+00 | 6.78E-01 | 7.23E+00 | 6.13E-01 | 6.17E-01 | 7.12E-01 |
| Pain in limb (Lower leg) | -6.89E-05 | 6.94E-04 | 9.21E-01 | 1.80E-03 | 2.75E-03 | 5.28E-01 | -1.47E-04 | 9.43E-04 | 8.76E-01 | 1.28E+01 | 3.04E-01 | 1.22E+01 | 2.70E-01 | 4.98E-01 | 6.80E-02 |
| Back pain | -3.00E-04 | 3.76E-03 | 9.36E-01 | 1.34E-02 | 9.17E-03 | 1.53E-01 | 5.74E-04 | 4.31E-03 | 8.94E-01 | 7.07E+01 | 1.40E-03 | 6.61E+01 | 3.14E-03 | 1.12E-01 | 5.00E-03 |
| Facial pain | -7.10E-04 | 1.21E-03 | 5.57E-01 | 1.89E-03 | 3.72E-03 | 6.16E-01 | -1.84E-03 | 1.47E-03 | 2.13E-01 | 3.83E+01 | 5.74E-02 | 3.74E+01 | 5.25E-02 | 4.67E-01 | 2.20E-02 |
| Headache | -2.00E-03 | 3.03E-03 | 5.09E-01 | -3.83E-03 | 7.63E-03 | 6.18E-01 | 3.53E-04 | 4.04E-03 | 9.30E-01 | 5.47E+01 | 4.92E-02 | 5.46E+01 | 3.99E-02 | 7.95E-01 | 6.00E-02 |
| Hip pain | -9.74E-05 | 2.23E-03 | 9.65E-01 | 3.28E-03 | 5.60E-03 | 5.61E-01 | 2.59E-03 | 2.98E-03 | 3.86E-01 | 4.75E+01 | 1.64E-01 | 4.70E+01 | 1.50E-01 | 5.14E-01 | 1.23E-01 |
| Knee pain | 3.13E-03 | 3.24E-03 | 3.35E-01 | -6.98E-03 | 8.36E-03 | 4.09E-01 | -6.28E-04 | 4.33E-03 | 8.85E-01 | 4.57E+01 | 1.55E-01 | 4.36E+01 | 1.80E-01 | 1.99E-01 | 1.96E-01 |
| Neck or shoulder pain | 2.26E-03 | 2.95E-03 | 4.45E-01 | 1.53E-02 | 7.08E-03 | 3.72E-02 | 6.46E-03 | 4.04E-03 | 1.09E-01 | 4.69E+01 | 1.81E-01 | 4.24E+01 | 2.88E-01 | 5.15E-02 | 2.35E-01 |
| Stomach or abdominal pain | -2.06E-03 | 2.02E-03 | 3.09E-01 | 2.36E-03 | 5.04E-03 | 6.42E-01 | -4.31E-03 | 2.65E-03 | 1.05E-01 | 4.96E+01 | 1.19E-01 | 4.84E+01 | 1.19E-01 | 3.45E-01 | 1.16E-01 |
| Pain all over the body | 6.59E-04 | 9.32E-04 | 4.79E-01 | -3.28E-03 | 2.37E-03 | 1.75E-01 | -8.87E-04 | 1.41E-03 | 5.29E-01 | 3.72E+01 | 5.08E-01 | 3.39E+01 | 6.15E-01 | 7.90E-02 | 3.09E-01 |

MR, Mendelian randomization; OR, odds ratio; CI, confidence intervals; IVW, inverse variance weighted.
