# Supplementary material for: Causal relationship between COVID-19 and chronic pain: A mendelian randomization study
Source: PLoS One. 2024 Jan 19;19(1):e0295982. doi: 10.1371/journal.pone.0295982 (PMC10798446; doi:10.1371/journal.pone.0295982)
Supplement: S3 Table — (DOCX) [file pone.0295982.s003.docx]

**Supplemental Table 3. MR estimates of associations between COVID-19 (hospitalized vs. not hospitalized) and bodily pain in various regions across different methods**

| Trait | IVW | | | MR Egger | | | Weighted median | | | Heterogeneity test | | | | Pleiotropy test | |
| --- | --- | --- | --- | --- | --- | --- | --- | --- | --- | --- | --- | --- | --- | --- | --- |
|  | β | se | p | β | se | p | β | se | p | IVW Q | p | MR‐Egger Q | p | MR‐Egger *p* | PRESSO *p* |
| Pain in joint | -3.56E-04 | 3.51E-04 | 3.11E-01 | 2.02E-03 | 4.40E-03 | 6.77E-01 | -4.13E-04 | 4.25E-04 | 3.31E-01 | 7.94E-01 | 9.39E-01 | 5.01E-01 | 9.19E-01 | 6.26E-01 | 9.34E-01 |
| Pain in joint (Lower leg) | -4.98E-04 | 5.34E-04 | 3.51E-01 | -1.55E-03 | 7.70E-03 | 8.53E-01 | -2.61E-04 | 5.59E-04 | 6.40E-01 | 8.27E+00 | 8.22E-02 | 8.22E+00 | 4.17E-02 | 8.99E-01 | 1.20E-01 |
| Low back pain | 6.32E-04 | 3.54E-04 | 7.41E-02 | -1.94E-03 | 2.53E-03 | 4.72E-01 | 3.23E-04 | 4.41E-04 | 4.64E-01 | 3.76E+00 | 8.07E-01 | 2.71E+00 | 8.44E-01 | 3.44E-01 | 8.38E-01 |
| Low back pain (Lumbar region) | -1.04E-04 | 3.48E-04 | 7.65E-01 | -6.05E-03 | 4.36E-03 | 2.60E-01 | 2.68E-04 | 4.44E-04 | 5.46E-01 | 2.95E+00 | 5.67E-01 | 1.08E+00 | 7.83E-01 | 2.65E-01 | 6.07E-01 |
| Pain in limb (Lower leg) | -4.48E-04 | 4.72E-04 | 3.42E-01 | -5.63E-03 | 5.74E-03 | 3.82E-01 | -3.62E-04 | 4.76E-04 | 4.46E-01 | 9.15E+00 | 1.03E-01 | 7.59E+00 | 1.08E-01 | 4.16E-01 | 1.32E-01 |
| Back pain | 1.15E-03 | 1.59E-03 | 4.70E-01 | -1.66E-03 | 3.99E-03 | 6.80E-01 | 1.65E-03 | 1.89E-03 | 3.81E-01 | 4.32E+01 | 1.33E-02 | 4.22E+01 | 1.24E-02 | 4.48E-01 | 5.98E-01 |
| Facial pain | 6.96E-05 | 4.45E-04 | 8.76E-01 | 1.71E-03 | 1.51E-03 | 2.75E-01 | 2.16E-05 | 5.99E-04 | 9.71E-01 | 1.10E+01 | 8.59E-01 | 9.67E+00 | 8.83E-01 | 2.73E-01 | 8.65E-01 |
| Headache | 6.27E-04 | 1.77E-03 | 7.24E-01 | 8.17E-03 | 4.16E-03 | 6.16E-02 | 2.31E-03 | 1.64E-03 | 1.59E-01 | 6.38E+01 | 3.07E-05 | 5.49E+01 | 3.27E-04 | 5.95E-02 | 4.42E-01 |
| Hip pain | 3.33E-04 | 8.78E-04 | 7.04E-01 | -4.98E-04 | 2.18E-03 | 8.21E-01 | 7.24E-04 | 1.21E-03 | 5.48E-01 | 1.80E+01 | 8.42E-01 | 1.78E+01 | 8.11E-01 | 6.81E-01 | 8.55E-01 |
| Knee pain | -9.38E-04 | 1.14E-03 | 4.10E-01 | -1.35E-03 | 2.82E-03 | 6.37E-01 | -4.57E-05 | 1.55E-03 | 9.77E-01 | 1.32E+01 | 9.73E-01 | 1.32E+01 | 9.63E-01 | 8.75E-01 | 9.75E-01 |
| **Neck or shoulder pain** | **2.85E-03** | **1.23E-03** | **2.00E-02** | **3.02E-04** | **3.02E-03** | **9.21E-01** | **1.21E-03** | **1.69E-03** | **4.74E-01** | **2.74E+01** | **3.34E-01** | **2.60E+01** | **3.51E-01** | **2.66E-01** | **3.54E-01** |
| Stomach or abdominal pain | 6.95E-04 | 7.83E-04 | 3.74E-01 | 1.86E-03 | 1.97E-03 | 3.54E-01 | 4.75E-04 | 1.11E-03 | 6.70E-01 | 2.53E+01 | 4.45E-01 | 2.49E+01 | 4.12E-01 | 5.24E-01 | 4.70E-01 |
| **Pain all over the body** | **8.19E-04** | **3.99E-04** | **4.01E-02** | **1.15E-03** | **9.93E-04** | **2.56E-01** | **1.14E-03** | **5.34E-04** | **3.34E-02** | **1.58E+01** | **9.20E-01** | **1.57E+01** | **8.99E-01** | **7.15E-01** | **9.18E-01** |

MR, Mendelian randomization; OR, odds ratio; CI, confidence intervals; IVW, inverse variance weighted.
