# Supplementary material for: Causal relationship between COVID-19 and chronic pain: A mendelian randomization study
Source: PLoS One. 2024 Jan 19;19(1):e0295982. doi: 10.1371/journal.pone.0295982 (PMC10798446; doi:10.1371/journal.pone.0295982)
Supplement: S4 Table — (DOCX) [file pone.0295982.s004.docx]

**Supplemental Table 4. MR estimates of associations between COVID-19 (very severe respiratory confirmed vs. normal population) and bodily pain in various regions across different methods**

| Trait | IVW | | | MR Egger | | | Weighted median | | | Heterogeneity test | | | | Pleiotropy test | |
| --- | --- | --- | --- | --- | --- | --- | --- | --- | --- | --- | --- | --- | --- | --- | --- |
|  | β | se | p | β | se | p | β | se | p | IVW Q | p | MR‐Egger Q | p | MR‐Egger *p* | PRESSO *p* |
| Pain in joint | 1.81E-05 | 1.85E-04 | 9.22E-01 | -1.74E-04 | 9.11E-04 | 8.51E-01 | -1.37E-05 | 2.63E-04 | 9.58E-01 | 1.48E+01 | 4.68E-01 | 1.47E+01 | 3.98E-01 | 8.33E-01 | 4.75E-01 |
| Pain in joint (Lower leg) | 1.52E-04 | 2.15E-04 | 4.78E-01 | 1.08E-04 | 1.09E-03 | 9.22E-01 | 2.66E-05 | 2.69E-04 | 9.21E-01 | 2.64E+01 | 9.09E-02 | 2.64E+01 | 6.75E-02 | 9.67E-01 | 7.90E-02 |
| Low back pain | -2.53E-04 | 2.36E-04 | 2.84E-01 | 5.00E-04 | 1.09E-03 | 6.54E-01 | -2.81E-04 | 3.29E-04 | 3.93E-01 | 1.26E+01 | 7.00E-01 | 1.21E+01 | 6.69E-01 | 4.91E-01 | 7.00E-01 |
| Low back pain (Lumbar region) | -2.16E-04 | 2.29E-04 | 3.44E-01 | -3.84E-04 | 1.13E-03 | 7.40E-01 | -4.44E-04 | 2.76E-04 | 1.07E-01 | 2.10E+01 | 1.03E-01 | 2.09E+01 | 7.44E-02 | 8.82E-01 | 1.18E-01 |
| Pain in limb (Lower leg) | -1.10E-04 | 1.81E-04 | 5.45E-01 | -1.16E-03 | 8.90E-04 | 2.11E-01 | -4.29E-04 | 2.53E-04 | 9.04E-02 | 1.57E+01 | 6.12E-01 | 1.43E+01 | 6.48E-01 | 2.46E-01 | 6.01E-01 |
| Back pain | 1.77E-03 | 9.74E-04 | 6.89E-02 | 3.54E-03 | 2.60E-03 | 1.80E-01 | 8.71E-04 | 1.39E-03 | 5.31E-01 | 5.77E+01 | 1.37E-01 | 5.70E+01 | 1.28E-01 | 4.66E-01 | 1.47E-01 |
| Facial pain | 5.99E-06 | 3.50E-04 | 9.86E-01 | 5.55E-04 | 1.10E-03 | 6.16E-01 | 1.75E-04 | 4.59E-04 | 7.02E-01 | 4.89E+01 | 7.40E-02 | 4.85E+01 | 6.39E-02 | 6.00E-01 | 8.00E-02 |
| Headache | 1.73E-03 | 1.01E-03 | 8.61E-02 | 2.62E-03 | 2.70E-03 | 3.37E-01 | 2.08E-03 | 1.20E-03 | 8.24E-02 | 7.34E+01 | 8.20E-03 | 7.32E+01 | 6.55E-03 | 7.24E-01 | 4.01E-01 |
| Hip pain | 9.02E-04 | 8.76E-04 | 3.03E-01 | 1.96E-03 | 2.35E-03 | 4.07E-01 | 1.22E-03 | 1.03E-03 | 2.35E-01 | 8.89E+01 | 2.15E-04 | 8.85E+01 | 1.70E-04 | 6.27E-01 | 9.53E-01 |
| Knee pain | 1.42E-03 | 9.61E-04 | 1.38E-01 | 2.39E-03 | 2.58E-03 | 3.59E-01 | 8.55E-04 | 1.27E-03 | 5.02E-01 | 6.37E+01 | 5.29E-02 | 6.34E+01 | 4.48E-02 | 6.89E-01 | 5.70E-02 |
| Neck or shoulder pain | -1.38E-04 | 9.76E-04 | 8.87E-01 | 7.81E-04 | 2.62E-03 | 7.67E-01 | -2.40E-05 | 1.34E-03 | 9.86E-01 | 6.21E+01 | 6.87E-02 | 6.19E+01 | 5.84E-02 | 7.06E-01 | 7.50E-02 |
| Stomach or abdominal pain | 3.61E-04 | 5.80E-04 | 5.34E-01 | 1.13E-03 | 1.55E-03 | 4.72E-01 | -5.28E-04 | 8.27E-04 | 5.23E-01 | 4.96E+01 | 3.72E-01 | 4.92E+01 | 3.44E-01 | 5.97E-01 | 3.83E-01 |
| **Pain all over the body** | **6.38E-04** | **3.17E-04** | **4.44E-02** | **2.12E-04** | **8.45E-04** | **8.03E-01** | **7.33E-04** | **4.60E-04** | **1.11E-01** | **5.46E+01** | **1.81E-01** | **5.42E+01** | **1.64E-01** | **5.89E-01** | **1.90E-01** |

MR, Mendelian randomization; OR, odds ratio; CI, confidence intervals; IVW, inverse variance weighted.
