# Supplementary material for: Causal relationship between COVID-19 and chronic pain: A mendelian randomization study
Source: PLoS One. 2024 Jan 19;19(1):e0295982. doi: 10.1371/journal.pone.0295982 (PMC10798446; doi:10.1371/journal.pone.0295982)
Supplement: S5 Table — (DOCX) [file pone.0295982.s005.docx]

**Supplemental Table 5. Details of SNPs extracted from exposure and F of them.**

| Exposure | SNP | effect_allele.exposure | other_allele.exposure | F |
| --- | --- | --- | --- | --- |
| COVID-19 (infection vs. normal population) | rs4916015 | A | G | 22.2468 |
|  | rs10798002 | C | A | 19.56032 |
|  | rs74069870 | C | T | 20.38916 |
|  | rs115333323 | A | C | 19.72172 |
|  | rs4971066 | G | T | 32.8158 |
|  | rs2126342 | G | C | 21.12759 |
|  | rs2271616 | T | G | 107.4254 |
|  | rs10936744 | T | C | 39.36797 |
|  | rs17078348 | G | A | 32.49438 |
|  | rs72682667 | G | C | 20.88255 |
|  | rs12189119 | C | T | 22.65754 |
|  | rs11133773 | A | G | 20.77062 |
|  | rs1979273 | A | T | 19.66268 |
|  | rs6554984 | A | G | 25.5817 |
|  | rs79568089 | T | C | 20.39708 |
|  | rs6911758 | C | G | 20.57191 |
|  | rs111837807 | C | T | 21.35049 |
|  | rs117204953 | C | T | 20.93417 |
|  | rs140434804 | T | C | 23.58464 |
|  | rs34766893 | A | T | 19.80524 |
|  | rs7786241 | G | A | 20.90422 |
|  | rs6583441 | T | A | 20.14614 |
|  | rs78913117 | T | G | 19.73929 |
|  | rs3850492 | A | G | 22.68797 |
|  | rs1853415 | A | G | 20.79612 |
|  | rs643434 | A | G | 100.3166 |
|  | rs138867328 | G | A | 20.90406 |
|  | rs75175435 | A | G | 19.70806 |
|  | rs7487474 | A | G | 26.48299 |
|  | rs757405 | A | T | 40.85893 |
|  | rs9524378 | A | G | 25.37287 |
|  | rs145175659 | A | G | 21.3621 |
|  | rs12593787 | T | C | 19.66741 |
|  | rs9922503 | C | A | 21.69108 |
|  | rs34266217 | C | G | 24.62176 |
|  | rs117538845 | A | G | 23.10547 |
|  | rs145459064 | T | C | 22.66214 |
|  | rs146390290 | C | G | 19.62809 |
|  | rs73000406 | A | C | 19.52027 |
|  | rs2109069 | A | G | 29.65095 |
|  | rs73054338 | T | G | 19.76865 |
|  | rs11672905 | A | G | 24.27297 |
|  | rs880278 | A | G | 22.88847 |
|  | rs12482060 | G | C | 34.64595 |
|  | rs139497823 | G | T | 19.98237 |
| COVID-19 (hospitalized vs. normal population) | rs56385977 | T | C | 20.74727 |
|  | rs10926975 | T | C | 19.78594 |
|  | rs41264915 | G | A | 29.20313 |
|  | rs12477217 | C | A | 21.71302 |
|  | rs78544343 | G | A | 23.20351 |
|  | rs45622439 | A | G | 20.76379 |
|  | rs140228296 | C | A | 21.40969 |
|  | rs1381109 | T | G | 23.4287 |
|  | rs35081325 | T | A | 240.128 |
|  | rs12519801 | T | C | 22.09354 |
|  | rs6888703 | G | T | 21.57987 |
|  | rs12519258 | A | G | 21.53379 |
|  | rs111837807 | C | T | 28.40853 |
|  | rs79204646 | T | C | 21.04997 |
|  | rs76828289 | C | T | 20.60868 |
|  | rs2897075 | T | C | 20.53884 |
|  | rs622568 | C | A | 34.80846 |
|  | rs62517724 | T | A | 19.74349 |
|  | rs17056406 | C | T | 22.30567 |
|  | rs138867466 | T | C | 26.47501 |
|  | rs505922 | C | T | 34.43305 |
|  | rs61622878 | T | C | 20.35273 |
|  | rs3940961 | A | G | 19.73397 |
|  | rs4310517 | G | C | 23.49481 |
|  | rs35189111 | T | C | 19.72956 |
|  | rs2282578 | T | A | 19.5851 |
|  | rs10860891 | A | C | 25.73488 |
|  | rs11835149 | T | C | 22.18076 |
|  | rs7953236 | C | T | 20.93961 |
|  | rs2660 | A | G | 35.97156 |
|  | rs113882976 | C | T | 21.85492 |
|  | rs80111688 | A | T | 21.01137 |
|  | rs111472920 | G | T | 20.68349 |
|  | rs74043130 | C | A | 20.13157 |
|  | rs117229619 | T | C | 23.36207 |
|  | rs112159047 | G | A | 25.2325 |
|  | rs1819040 | A | T | 28.43622 |
|  | rs17069506 | C | T | 27.29892 |
|  | rs8096865 | T | C | 21.08255 |
|  | rs2109069 | A | G | 57.77863 |
|  | rs13050728 | C | T | 69.55046 |
| COVID-19 (hospitalized vs. not hospitalized) | rs12126146 | G | A | 20.43228 |
|  | rs838102 | A | G | 20.82551 |
|  | rs56215822 | T | C | 20.11054 |
|  | rs140636356 | T | A | 19.86667 |
|  | rs72893671 | A | T | 34.12152 |
|  | rs77838749 | C | G | 27.06562 |
|  | rs111242223 | C | T | 20.79285 |
|  | rs78191176 | C | T | 24.10354 |
|  | rs1444887 | C | T | 20.40536 |
|  | rs61543598 | C | T | 20.12925 |
|  | rs112863318 | A | G | 19.61541 |
|  | rs7357033 | A | G | 20.81596 |
|  | rs77718271 | T | C | 25.88177 |
|  | rs74669707 | A | G | 21.77687 |
|  | rs78498740 | A | G | 20.88098 |
|  | rs190583048 | C | T | 24.39247 |
|  | rs79140987 | G | A | 20.53719 |
|  | rs56220218 | A | G | 21.07784 |
|  | rs79628038 | C | T | 20.2585 |
|  | rs78776847 | A | G | 19.62066 |
|  | rs55647725 | G | A | 26.2392 |
|  | rs7193153 | C | A | 19.86469 |
|  | rs143821051 | T | C | 21.26481 |
|  | rs7225002 | G | A | 24.6059 |
|  | rs77046349 | T | C | 20.80678 |
|  | rs73552634 | T | C | 22.86259 |
|  | rs77866768 | T | G | 20.91533 |
|  | rs7351246 | T | C | 22.71071 |
| COVID-19 (very severe respiratory confirmed vs. normal population) | rs111508230 | T | C | 22.0807 |
|  | rs340850 | G | T | 21.12287 |
|  | rs4076440 | G | A | 24.05787 |
|  | rs9287218 | C | A | 25.73274 |
|  | rs113488799 | C | T | 19.73309 |
|  | rs73980984 | G | T | 20.35829 |
|  | rs1465533 | C | A | 26.18271 |
|  | rs35081325 | T | A | 197.9819 |
|  | rs28562934 | T | C | 23.13953 |
|  | rs9872763 | G | A | 19.6364 |
|  | rs13080258 | C | A | 21.46747 |
|  | rs202172202 | T | C | 20.64469 |
|  | rs372783206 | A | G | 21.2281 |
|  | rs141982888 | C | G | 20.73245 |
|  | rs7654748 | C | T | 19.76303 |
|  | rs79833209 | T | C | 22.39646 |
|  | rs114969787 | T | C | 21.46887 |
|  | rs6901969 | C | T | 19.66475 |
|  | rs111837807 | C | T | 47.44426 |
|  | rs2237698 | T | C | 35.60826 |
|  | rs77406469 | T | C | 24.75773 |
|  | rs622568 | C | A | 37.23955 |
|  | rs36932 | A | G | 21.24508 |
|  | rs4389947 | A | G | 25.01487 |
|  | rs3735891 | G | C | 22.85857 |
|  | rs6478109 | G | A | 26.67961 |
|  | rs10124390 | A | C | 21.65482 |
|  | rs550057 | T | C | 28.02801 |
|  | rs7080472 | T | G | 19.60486 |
|  | rs118052809 | A | T | 19.92198 |
|  | rs116637605 | T | C | 20.59527 |
|  | rs78123456 | C | T | 20.72532 |
|  | rs2443005 | C | T | 29.46874 |
|  | rs10771479 | A | G | 21.27766 |
|  | rs2384074 | T | C | 49.38288 |
|  | rs2733839 | C | T | 21.5085 |
|  | rs10860891 | A | C | 36.36412 |
|  | rs117232645 | A | G | 22.16116 |
|  | rs4942151 | T | C | 19.63713 |
|  | rs9577175 | T | C | 24.38354 |
|  | rs59030069 | T | C | 19.79014 |
|  | rs11634857 | A | G | 25.28916 |
|  | rs11658357 | A | T | 20.88492 |
|  | rs77534576 | T | C | 37.63609 |
|  | rs2109069 | A | G | 83.58532 |
|  | rs45524632 | A | C | 23.75756 |
|  | rs1974792 | G | A | 25.22336 |
|  | rs76053670 | A | G | 19.88148 |
|  | rs117210925 | T | C | 19.6815 |
|  | rs13050728 | C | T | 49.09662 |
|  | rs5767981 | G | A | 20.85376 |
